# Cricket protein hydrolysate as a sustainable functional ingredient in dog diets: Effects on palatability, health parameters, and antioxidant shelf-life stability

**DOI:** 10.14202/vetworld.2025.2678-2688

**Published:** 2025-09-11

**Authors:** Nuttawadee Saejiem, Chaiyapoom Bunchasak, Kanokporn Poungpong

**Affiliations:** 1 Department of Animal Science, Faculty of Agriculture, Kasetsart University, Bangkok, 10900, Thailand; 2 Department of Physiology, Faculty of Medicine, Kasetsart University, Bangkok, 10900, Thailand

**Keywords:** amino acid profile, insect-based protein, oxidative stability, palatability, sustainable pet food

## Abstract

**Background and Aim:**

Insect-derived proteins are gaining attention as sustainable pet food ingredients, but the use of cricket protein hydrolysate (CPH) in canine diets remains underexplored. This study evaluated the effects of CPH on diet palatability, physiological responses, and antioxidant potential for shelf-life extension in commercial dog food.

**Materials and Methods:**

Thirty-two healthy adult dogs were assigned to four diets containing 0%, 2%, 4%, or 6% CPH for a 30-day feeding trial. Palatability was assessed through a two-bowl preference test, while biochemical, hematological, and fecal parameters were measured pre- and post-trial. Antioxidant efficacy was evaluated by monitoring acid value (AV) and peroxide value (PV) during accelerated storage (55°C for 46 days, simulating 12 months). Nutritional adequacy was confirmed through proximate and amino acid analysis.

**Results:**

The 2% CPH diet significantly improved palatability, with a 57% increase in intake compared to control (p < 0.05), whereas higher inclusions (4% and 6%) reduced acceptance due to bitterness from hydrophobic peptides. All health parameters remained within reference ranges, though the 6% CPH diet lowered serum glucose (87.0 vs. 112.0 mg/dL; p < 0.001) and increased blood urea nitrogen (11.0 mg/dL; p = 0.0023). Antioxidant activity increased with CPH level, with 6% CPH reducing PV by 33% after 46 days (p < 0.05). CPH lacked certain essential amino acids, notably tryptophan, requiring complementary protein supplementation.

**Conclusion:**

CPH is a multifunctional ingredient that can enhance palatability and oxidative stability in dog diets at moderate inclusion (2%). High inclusion levels improve antioxidant capacity but may impair sensory acceptance and alter metabolic markers. Long-term safety, allergenicity, and flavor-masking strategies warrant further study.

## INTRODUCTION

In recent years, insect-based pet foods have attracted growing interest as sustainable alternatives to conventional plant- and animal-derived ingredients, providing high-quality protein along with essential micronutrients such as B-complex vitamins, zinc, copper, and phosphorus [1]. Beyond their rich nutrient profile, insects offer a favorable fatty acid composition and a markedly lower environmental footprint compared with traditional livestock sources [2, 3].

Among edible insect species, crickets (*Acheta domesticus*) stand out for their exceptional nutrient density, requiring smaller quantities than chicken or fish to meet equivalent protein needs [1]. Nonetheless, certain limitations hinder their broader adoption. Substituting chicken meal with cricket meal can reduce digestibility and increase dietary fiber content due to the presence of chitin, an indigestible structural component for monogastric animals [4]. Moreover, cricket-derived ingredients may emit undesirable odors, negatively impacting palatability, a key factor influencing both pet consumption and owner acceptance [5].

Enzymatic hydrolysis presents a viable strategy to address these issues. The production of cricket protein hydrolysate (CPH) can enhance digestibility by breaking down chitin and other antinutritional factors, while simultaneously generating flavor-enhancing peptides [6]. Hydrolysates are also capable of minimizing off-odors (e.g., fishy notes) and intensifying umami flavor through the release of bioactive peptides such as asparagine–proline [7, 8]. In addition, crickets exhibit inherent antioxidant potential [9], positioning CPH as a promising natural preservative in lipid-rich pet food formulations to mitigate rancidity and extend shelf life [10].

Although insect-based proteins, particularly cricket (*A. domesticus*) meal, have been increasingly recognized as sustainable and nutrient-rich ingredients for companion animal diets, most existing studies have focused on whole cricket meal or crude cricket powder as protein sources. These studies primarily address nutrient digestibility, basic health outcomes, and environmental sustainability, but they often overlook critical sensory and functional aspects, such as palatability and oxidative stability, which are key determinants of commercial adoption in the pet food industry. The presence of chitin and other antinutritional factors in whole cricket meal can reduce digestibility and alter feed texture, while off-odors and bitter notes can negatively influence feed acceptance.

Enzymatic hydrolysis has been successfully applied to other protein sources to enhance digestibility, release bioactive peptides, and improve sensory qualities; however, its application to crickets remains largely unexplored in the context of canine nutrition. There is limited evidence on how CPH affects palatability, nutrient utilization, and long-term health markers in dogs. Furthermore, while crickets are known to possess antioxidant compounds, no comprehensive studies have evaluated the potential of CPH as a natural preservative in lipid-rich dog food formulations under realistic storage conditions. Addressing these gaps is essential to determine the functional value of CPH beyond its nutritional profile and to establish safe and optimal inclusion levels for commercial pet food production.

This study aimed to investigate the potential of CPH as a multifunctional ingredient in premium commercial dog diets. Specifically, the objectives were to:

1. Evaluate the palatability of CPH-supplemented diets using a standardized two-bowl preference test to determine the inclusion level that maximizes feed acceptance.
2. Assess physiological and metabolic effects of CPH supplementation by monitoring hematological and biochemical parameters, ensuring inclusion levels remain within safe and nutritionally adequate ranges.
3. Examine antioxidant efficacy of CPH in enhancing oxidative stability of dog food during accelerated storage, simulating long-term shelf life.
4. Characterize the amino acid profile of CPH and compare it with Association of American Feed Control Officials (AAFCO) nutritional requirements to identify potential deficiencies and guide complementary formulation strategies.

By integrating nutritional, sensory, and preservative evaluations, this research seeks to provide a comprehensive understanding of CPH’s applicability as a sustainable, functional, and commercially viable ingredient for the pet food industry.

## MATERIALS AND METHODS

### Ethical approval

All experimental procedures were approved by the Institutional Animal Care and Use Committee of Kasetsart University, Thailand (Approval No. ACKU67-AGR-012).

### Study period and location

The study was conducted from June 2022 to December 2023. Proximate analysis was conducted at Kasetsart University, Thailand; diet preparation and shelf-life evaluation were performed at Absolute Nutrition Co., Ltd., Thailand; and the dog feeding trial took place at the Animal Quarantine Center, Phetchaburi, Thailand.

### Preparation of CPH

Adult *A. domesticus* crickets were sourced from Chutikarn Cricket Farm, Sukhothai Province, Thailand. The crickets were processed into a fine powder by baking and grinding. To prepare the hydrolysate, 200 g of cricket powder was homogenized with 500 mL of distilled water using a magnetic stirrer for 2 min to obtain a uniform slurry. The slurry was pasteurized at 90°C for 15 min, adjusted to pH 7.7 with 5 M NaOH (Sigma-Aldrich, USA), and equilibrated at 50°C. Alcalase (Sunson Biotechnology, China) was then added at a 5% (w/w) enzyme-to-substrate ratio, and the mixture was hydrolyzed for 60 min. The pH was subsequently adjusted to 6.4 using 5 M HCl (Sigma-Aldrich) before incubation with Flavourzyme (Sunson Biotechnology, China) at the same enzyme-to-substrate ratio for an additional 60 min at 55°C. The enzymatic reaction was terminated by boiling at 100°C for 5 min, cooling to 4°C, and centrifuging at 4,000 × *g* for 15 min in a refrigerated centrifuge (Model 5810R, Eppendorf, Germany). The resulting supernatant was collected for amino acid analysis and later incorporated into the experimental diets.

### Formulation of experimental diets

Four experimental dog food formulations were developed to meet the AAFCO nutritional guidelines for adult dogs: A control diet (0% CPH) and three CPH-supplemented diets containing 2%, 4%, or 6% CPH. All diets were manufactured by Absolute Nutrition (Thailand) and certified by the Department of Livestock Development. Following formulation, diets were dried in a hot-air oven at 70°C for 9 h and cut into uniform 1 × 1 cm pieces for consistency.

### Animal selection

A total of 32 healthy adult dogs (aged 1–7 years; body weight [BW] 8.0 ± 2.5 kg), representing medium-sized breeds and balanced by sex, were randomly assigned to the four dietary treatments (n = 8 per group). All dogs were clinically healthy, non-pregnant, non-lactating, regularly vaccinated, and housed individually in the same facility throughout the trial. Before enrollment, each dog was confirmed to be free from chronic disease and maintained on a standard commercial diet.

### Experimental design and feeding protocol

The study employed a completely randomized design with four dietary treatments (0%, 2%, 4%, and 6% CPH) and eight dogs per treatment for a 30-day feeding period. Dogs underwent a 3-day adaptation period before the trial, transitioning from their regular diet to the assigned test diet. Feeding occurred 3 times daily (08:00, 12:00, and 16:00). Daily feed allotments were calculated based on metabolizable energy (ME) requirements per AAFCO guidelines, with an additional 30% allowance to encourage *ad libitum* intake during palatability testing. BW and daily feed intake (offered minus refused) were measured at baseline and on day 30 to determine consumption and weight change.

### Palatability assessment

Palatability was assessed using the standard two-bowl preference test, a validated method in canine nutrition research. Dogs were offered two diets simultaneously for 30 min each day at 16:00, with bowl positions alternated daily to eliminate side bias. Two metrics were recorded: (1) First-choice selection and (2) total intake, providing a comprehensive measure of dietary preference.

### Fecal sample collection and analysis

On day 30, total fecal output was collected for each dog. Fecal consistency was scored on a 0–5 scale (0 = None, 5 = Hard, dry, and crumbly). Samples were analyzed for dry matter and moisture content.

### Blood sampling and analysis

Blood samples were collected from the jugular vein at baseline and on day 30. For hematological analysis, 2 mL of whole blood was collected in EDTA tubes. For biochemical analysis, 3 mL of blood was collected into serum separator tubes, allowed to clot for 30 min, and centrifuged at 4,000 × *g* for 15 min at 4°C (Eppendorf, Model 5810R, Germany). Serum was harvested and stored at 4°C until analysis.

### Simulation of oxidative stability and shelf-life

To simulate shelf-life conditions, 1 kg of each treatment diet was packaged in storage bags with a pinhole to permit air exchange and stored at 55°C in a dark-cycle incubator (Memmert, Model IF55, Germany). Storage durations were 0, 23, and 46 days, representing 0, 6, and 12 months, respectively. Lipid oxidation was measured using AOCS official methods. Peroxide value (PV) was determined according to methods Ab 5-49 and Cd 8b-90 and expressed as milliequivalents (meq) peroxide/kg extracted fat. Acid value (AV) was measured using methods Cd 3d-63 and Ab 5-49, expressed as mg potassium hydroxide (KOH)/g extracted oil.

### Statistical analysis

Data analysis was conducted using the Statistical Analysis System (SAS) software (version 9.4; SAS Institute Inc., USA). Statistical significance was set at p < 0.05. Data are presented as mean ± standard error of the mean. Normality and homogeneity of variance assumptions were verified before analysis. Biochemical and hematological parameters were analyzed using a mixed model, with sex and cage as blocking factors and cage as the experimental unit. Orthogonal polynomial contrasts (linear, quadratic, and cubic) were applied to evaluate dose-response trends. Palatability data were analyzed through one-way analysis of variance, and treatment differences were determined using least squares means with Duncan’s multiple range test.

## RESULTS AND DISCUSSION

### Amino acid composition of CPH

The amino acid composition of CPH derived from *A. domesticus* was compared with the AAFCO minimum requirements for adult maintenance and growth/reproduction in dogs (Table 1). CPH showed increased concentrations of several amino acids, especially arginine (0.92% vs. 0.43%), aspartic acid (0.18% vs. 0.13%), and glutamine (0.10% vs. 0.04%), compared with the unprocessed cricket solution, as a result of enzymatic hydrolysis releasing bound amino acids [8].

**Table 1:** Amino acid profiles of cricket solution and CPH compared with the AAFCO minimum nutritional requirements for dogs.

Amino acid (%)	Growth and reproduction (min.)	Adult maintenance (min.)	Cricket solution	CPH
Arginine	1.00	0.51	0.43	0.92
Histidine	0.44	0.19	0.12	0.13
Isoleucine	0.71	0.38	0.03	0.06
Leucine	1.29	0.68	0.13	0.16
Lysine	0.90	0.63	0.09	0.12
Methionine	0.35	0.33	0.12	0.13
Methionine + cysteine	0.70	0.65	0.14	0.16
Phenylalanine	0.83	0.45	0.06	0.06
Phenylalanine + tyrosine	1.30	0.74	0.10	0.11
Threonine	1.04	0.48	0.13	0.18
Tryptophan	0.20	0.16	0.00	0.00
Valine	0.68	0.49	0.08	0.11
Glutamine	N/A	N/A	0.04	0.10
Aspartic acid	N/A	N/A	0.13	0.18

AAFCO = Association of American Feed Control Officials, CPH = Cricket protein hydrolysate, CI = Confidence interval, Min. = Minimum, N/A = Not applicable

Despite these enhancements, CPH did not meet the AAFCO minimum requirements for several essential amino acids, including histidine (0.13% vs. 0.19%), isoleucine (0.06% vs. 0.38%), and leucine (0.16% vs. 0.68%). This finding highlights the necessity of combining CPH with complementary protein sources to ensure nutritional adequacy in canine diets.

The absence of detectable tryptophan in CPH (Table 1) is particularly critical, given its essential role in serotonin synthesis and nitrogen balance in dogs [11]. To address this, tryptophan-rich ingredients should be incorporated in future formulations; for instance, blending CPH with 10%–15% poultry by-product meal would meet AAFCO minimums, correct the amino acid profile, and improve both digestibility and palatability. This strategy is consistent with previous reports indicating that amino acid complementation in monogastric diets is required for insect-based proteins [12].

### Functional properties beyond nutrition

CPH demonstrated potential as a multifunctional ingredient beyond its nutritional profile. The high concentrations of hydrophobic amino acids (e.g., valine, leucine, and phenylalanine) and sulfur-containing residues (e.g., methionine and cysteine) in CPH are consistent with its antioxidant activity.

Methionine (0.13%) and cysteine (contributing to Met-Cys levels of 0.16%) are recognized for their roles in reducing oxidative rancidity through decarboxylation to form 3-(methylthio) propylamine, a compound with synergistic antioxidant properties, particularly under high-temperature conditions [13]. These properties support the potential of CPH as a multifunctional ingredient in fat-rich pet food formulations that enhance both flavor and oxidative stability.

Peptides, such as asparagine-proline, generated during enzymatic hydrolysis, are known for their umami-enhancing effects [6], which, further, support the use of CPH in improving feed palatability, which is an important factor in pet owner adherence [5].

### Nutritional response and palatability

The inclusion of CPH in dog diets significantly affected feed intake, BW gain (BWG), and nutrient composition (Table 2). We observed a dose-dependent decrease in feed intake, with the lowest intake occurring in the 6% CPH group (139.99 ± 1.97 g/day) compared to the control (215.97 ± 2.45 g/day; p < 0.05). This reduced intake at higher CPH levels suggests decreased palatability, likely due to the formation of free amino acids and hydrophobic peptides during enzymatic hydrolysis [14].

**Table 2:** Effect of CPH replacement on nutrient composition, feed intake, and growth performance in dog diets.

Item	CPH inclusion level (%)			

	0	2	4	6
ME (kcal/kg)	2,276.05 ± 2.82^a^	2,461.75 ± 0.80^b^	2,449.20 ± 1.21^b^	2,458.70 ± 1.00^b^
CP (%)	23.39 ± 0.38^a^	24.11 ± 0.13^b^	23.68 ± 0.12^a^	23.80 ± 0.28^a^
CF (%)	14.00 ± 0.28^a^	13.80 ± 0.11^ab^	13.74 ± 0.09^b^	13.84 ± 0.18^ab^
CFb (%)	0.19 ± 0.02^a^	0.17 ± 0.01^b^	0.20 ± 0.01^a^	0.20 ± 0.01^a^
Ash (%)	3.66 ± 0.03^a^	3.67 ± 0.04^a^	3.76 ± 0.09^b^	3.63 ± 0.04^a^
Mo (%)	13.21 ± 0.20^a^	13.76 ± 0.37^b^	13.37 ± 0.15^a^	13.60 ± 0.34^ab^
Feed intake (g) (as fed/day)	215.97 ± 2.45^a^	183.98 ± 5.05^b^	187.62 ± 4.83^b^	139.99 ± 1.97^c^
ME intake (kcal/kg)	419.54	452.91	459.51	344.19
BWG (kg)	0.05	1.17	−1.01	−0.43
Fecal score	3.46	3.57	3.21	3.95

^a,b,c^Within a row with no common letter differs significantly (p < 0.05). Values are presented as mean ± standard error of the mean. CPH = Cricket protein hydrolysate, ME = Metabolizable energy, CP = Crude protein, CF = Crude fat, CFb = Crude fiber, Ash = Crude ash, Mo = Moisture, BWG = Body weight gain

Interestingly, the 2% CPH group achieved the highest BWG (1.17 kg), while negative BWG was observed at 4% (−1.01 kg) and 6% (−0.43 kg). This indicates that the inclusion level of 2% provided a favorable balance between nutrient absorption and palatability, whereas higher levels led to feed aversion and subsequent weight loss.

Proximate analysis showed an increase in ME with CPH inclusion, reflecting the high protein bioavailability of the hydrolysate. The crude protein (CP) content peaked at 24.11% in the 2% CPH diet but did not increase further at higher inclusion levels, likely due to a dilution effect from other ingredients. Moisture and ash content remained relatively consistent across treatments, although ash levels increased slightly at 4% CPH (3.76%; p < 0.05), potentially due to CPH’s mineral input.

The fecal scores across all groups were excellent (3.21–3.95), indicating that CPH replacement caused no significant gastrointestinal disturbances, which is consistent with previous reports on the high digestibility of insect-based proteins in dogs [13].

### Two-bowl preference test findings

**Figure 1:**
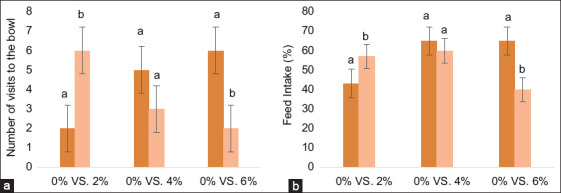
Palatability of dog diets with varying cricket protein hydrolysate inclusion levels (0%, 2%, 4%, and 6%). Panels show (a) The number of visits to each bowl during the preference test and (b) the average daily feed intake percentage for each diet over a 3-day period (n = 8 dogs per group). Data are presented as mean ± standard error of the mean. Bars with different letters (a and b) are significantly different (p < 0.05).

In a two-bowl preference test, dogs confirmed the palatability trends by showing a strong preference for the 2% CPH diet (Figure 1). This group had a significantly higher first-choice selection rate and a greater average intake (57.00%) than the control group. The relatively higher crude fat content of the control diet (Table 2) may have contributed to its consistent acceptance, as dietary lipids serve as flavor carriers and enhance palatability [15].

Conversely, the 4% and 6% CPH diets were less preferred. This dose-dependent response is consistent with the literature on protein hydrolysates: Low-to-moderate levels of protein hydrolysates can improve flavor by releasing umami-enhancing peptides (e.g., glutamic acid), whereas excessive hydrolysis produces bitter hydrophobic peptides that reduce feed acceptance [5, 16].

Hydrolyzed proteins may improve nutrient absorption and stimulate satiety-related hormones, such as cholecystokinin and peptide YY, which could help regulate food intake [17]. The success of the 2% CPH diet suggests that an optimal balance was struck between these flavor and off-flavor-generating compounds.

In conclusion, a 2% CPH inclusion level appears to be a practical and effective strategy for boosting the nutritional value of canine diets without compromising palatability. The negative sensory impact at higher concentrations highlights a key challenge, similar to issues seen in plant protein hydrolysates [6].

Future research should investigate strategies such as encapsulation, the use of flavor enhancers, or blending CPH with other protein sources to mask bitterness to utilize CPH at higher inclusion rates [18]. Further studies should also examine the long-term effects of CPH on gastrointestinal health and satiety to fully optimize its application in pet food.

### Blood biochemistry and hematological analysis

Dogs consuming diets supplemented with CPH for 30 days exhibited statistically significant but clinically acceptable metabolic and physiological changes (Table 3). All measured parameters remained within normal reference ranges, with dose-dependent effects indicating both benefits and areas for consideration.

**Table 3:** Effects of CPH replacement in dog diets on blood biochemical parameters related to general metabolism, kidney function, and liver function.

Item	CPH inclusion level (%)				Reference values	SEM	p-value

	0	2	4	6			
General metabolism							
Glucose (mg/dL)	112.00^a^	118.00^b^	113.00^a^	87.00^c^	68–104	1.15	0.0001^C^
Kidney function							
BUN (mg/dL)	4.00^a^	9.00^b^	10.00^bc^	11.00^c^	9–26	0.79	0.0023^Q^
Creatinine (mg/dL)	0.51^a^	0.46^b^	0.42^c^	0.38^d^	0.6–1.4	0.01	0.0001^L^
Liver function							
Total protein (g/dL)	5.50^a^	5.90^b^	5.70^b^	5.50^a^	5.5–7.2	0.08	0.0011^Q^
Albumin (g/dL)	2.60^a^	2.60^a^	2.40^b^	2.30^b^	3.2–4.1	0.10	0.0028^L^
Globulin (g/dL)	2.90^a^	3.30^b^	3.30^b^	3.20^b^	1.9–3.7	0.10	0.0083^L^
ALP (U/L)	221.00^a^	135.00^b^	125.00^c^	112.0^d^	7–115	1.00	0.0001^C^
AST (U/L)	26.00^a^	29.00^b^	37.00^c^	45.00^d^	18–56	0.91	0.0022^Q^
ALT (U/L)	22.00^a^	22.00^ab^	21.00^b^	20.00^b^	17–95	0.33	0.0369^Q^
GGT (U/L)	5.00^a^	4.00^b^	4.00^b^	4.00^b^	0–8	0.41	0.0278^Q^
Total bilirubin (mg/dL)	0.04^a^	0.08^b^	0.07^c^	0.06^d^	0–0.2	1.33	0.0015^C^
Cholesterol (mg/dL)	214.00^a^	224.00^b^	218.00^b^	210.00^c^	136–392	3.00	0.0002^C^
Triglyceride (mg/dL)	85.00^a^	50.00^b^	50.00^b^	56.00^c^	23–102	3.00	0.0002^C^

^a,b,c,d^Within a row with no common letter differs significantly (p < 0.05). The letters L, Q, and C on the p-value indicate a significant linear, quadratic, or cubic dose-response relationship, respectively. CPH = Cricket protein hydrolysate, SEM = Standard error of the mean, GGT = Gamma-glutamyl transferase, ALT = Alanine transaminase, AST = Aspartate transaminase, ALP = Alkaline phosphatase, BUN = Blood urea nitrogen

At 6% CPH, serum glucose levels were significantly decreased (87.00 vs. 112.00 mg/dL in controls; p < 0.001), suggesting improved insulin sensitivity and energy use. This effect is likely mediated by low-molecular-weight peptides (<3 kDa) that inhibit α-glucosidase and dipeptidyl peptidase 4 and activate the PI3K/Akt and AMP-activated protein kinase pathways [19]. Antioxidant peptides may also protect pancreatic β-cells from oxidative stress, supporting glucose regulation [20].

Blood urea nitrogen (BUN) levels increased with higher CPH (6%: 11.00 mg/dL; p = 0.0023), while creatinine decreased (0.38 mg/dL; p < 0.001), implying increased nitrogen turnover without renal burden. This inverse BUN-creatinine trend may result from enhanced amino acid metabolism and urea cycle activity, driven by branched-chain amino acids and arginine- or ornithine-containing peptides [13, 21].

At 2% CPH, the dogs gained weight (+1.17 kg) with stable glucose, triglyceride, and cholesterol levels, indicating energy sufficiency. Conversely, the 4% and 6% CPH diets were associated with weight loss (1.01 kg and 0.43 kg, respectively), reduced feed consumption, and metabolic changes, including lower glucose, triglycerides, and creatinine levels and elevated BUN, suggesting that weight loss is driven by reduced energy intake rather than metabolic impairment.

The liver function indicators showed variable responses. Total serum protein was highest at 2% CPH (5.90 g/dL; p = 0.0011), likely reflecting enhanced protein bioavailability, whereas albumin decreased at 6% CPH (2.30 g/dL; p = 0.0028), potentially suggesting mild hepatic stress.

ALP activity declined linearly with increasing CPH (6%: 112.04 U/L; p < 0.001), potentially indicating reduced oxidative stress and improved hepatic function through Nrf2-mediated antioxidant activity [9, 19, 21]. Aspartate transaminase (AST) levels quadratically increased (6%: 45.00 U/L; p = 0.0022) but remained within normal limits. This pattern points to hepatic adaptation rather than dysfunction, which is consistent with observations of temporary AST elevations in dogs on other high-protein or hydrolyzed protein diets [22–24].

The lipid profiles were also affected. At 2% CPH, cholesterol increased (224.00 mg/dL; p < 0.001), likely due to cricket fat content, while triglycerides decreased (50.00 mg/dL; p < 0.001), possibly from increased lipid catabolism associated with reduced intake [15, 25]. Mineral levels (calcium and phosphorus) remained within physiological ranges, suggesting no adverse impact on mineral balance. These findings are consistent with those of rodent studies showing that insect-based diets improve lipid profiles without adversely affecting liver function [26].

### Immune and hematological changes

In addition to these metabolic markers, hematological analysis demonstrated dose-dependent immunological changes (Table 4). Marked reductions in white blood cell (WBC) counts (e.g., 6% CPH: 12.3×10³/μL vs. control: 23.0×10³/μL; p < 0.01) suggest systemic immune modulation, possibly driven by anti-inflammatory peptides in CPH that suppress cytokines such as tumor necrosis factor-alpha and interleukin-6 [9, 27]. Although WBC levels remained within the normal range, the extent of reduction may indicate either an anti-inflammatory benefit or potential immune suppression; further research on cytokine expression and lymphocyte profiles is warranted to clarify these effects.

**Table 4:** Hematological profiles of dogs fed diets replaced with CPH.

Item	CPH inclusion level (%)				Reference values	SEM	p-value

	0	2	4	6			
WBC (10^3^/mm^3^)	23.00^a^	13.00^b^	12.00^c^	12.30^b^	5.00–14.00	0.58	0.0097^C^
RBC (10^6^/mm^3^)	5.04^a^	6.03^b^	6.05^b^	5.98^b^	4.95–7.87	0.09	0.0592^C^
Hemoglobin (g/dL)	11.20^a^	12.60^b^	12.50^b^	12.30^c^	11.90–18.90	0.01	0.0016^C^
Hematocrit (%)	34.40^a^	38.50^b^	38.00^c^	36.90^d^	35.00–57.00	0.02	0.0001^C^
MCV (fL)	68.30^a^	63.80^b^	62.60^c^	61.70^d^	66.00–77.00	0.01	0.0001^C^
MCH (pg)	22.20^a^	20.90^b^	20.70^b^	20.60^b^	21.00–26.20	0.01	0.0001^C^
MCHC (g/dL)	32.60^a^	32.70^a^	33.00^b^	33.30^c^	32.00–36.30	0.01	0.0098^C^
Platelets (10^3^/mm^3^)	422.00^a^	206.00^b^	320.00^c^	476.00^d^	211.00–621.00	2.67	0.0001^C^
Monocytes (%)	6.00^a^	10.00^b^	6.00^a^	12.00^c^	2.00–10.00	1.25	0.0001^Q^
Eosinophils (%)	11.00^a^	9.00^b^	15.00^c^	21.00^d^	0.00–9.00	1.00	0.0023^C^
Neutrophils (%)	63.00^a^	58.00^a^	43.00^b^	28.00^c^	58.00–85.00	2.83	0.0129^C^
Lymphocytes (%)	20.00^a^	32.00^b^	36.00^c^	39.00^c^	8.00–21.00	3.50	0.0075^Q^

^a,b,c,d^Within a row with no common letter differ significantly (p < 0.05). The letters L, Q, and C on p-value indicate a significant linear, quadratic, or cubic dose-response relationship, respectively. CPH = Cricket protein hydrolysate, SEM = Standard error of the mean, WBC = White blood cell, RBC = Red blood cell, MCV = Mean corpuscular volume, MCH = Mean corpuscular hemoglobin, MCHC = Mean corpuscular hemoglobin concentration

Conversely, elevated hemoglobin concentrations (6% CPH: 12.30 g/dL vs. control: 11.20 g/dL; p < 0.01) indicate enhanced erythropoiesis, with these findings suggesting a role for the bioavailability of iron and B-vitamins from crickets [28].

Increased eosinophil percentages observed in the 6% CPH group (21.00% vs. 11.00% in controls; p < 0.01) may indicate allergic sensitization to arthropod-derived proteins. This finding aligns with reports of immunogenic cross-reactivity between insect-derived and crustacean tropomyosin, a conserved muscle protein capable of eliciting immunoglobulin E-mediated responses [29, 30].

Future research should incorporate extended observation periods, comprehensive immunological profiling, and gut microbiota analysis to elucidate whether microbial alterations modulate eosinophilic responses or immune tolerance. Recognizing these immunogenic risks is essential for establishing safety guidelines.

Nonlinear variations in platelet counts (4% CPH: 320.00×10³/μL vs. 6%: 476.00×10³/μL; p < 0.01), along with changes in mean corpuscular volume and mean corpuscular hemoglobin values, could also indicate underlying oxidative stress or disruptions in iron metabolism [31].

The decrease in WBC subtypes, namely, neutrophils, lymphocytes, and monocytes, corresponds with previous findings on hydrolyzed plant proteins, where bioactive peptides were shown to suppress both innate and adaptive immune responses to reduce inflammation [6]. However, sustained suppression of immune cell populations may increase susceptibility to infection, warranting caution regarding long-term consumption of CPH.

Elevations in eosinophils and basophils, along with fluctuating platelet counts, suggest a dual effect of CPH: It may reduce chronic inflammation and potentially trigger allergic or T helper 2-biased immune responses [32].

### Summary of health outcomes

In summary, this 30-day study demonstrates that CPH exerts complex, dose-dependent effects. Moderate inclusion (2%–4%) offered metabolic benefits and potential anti-inflammatory activity without adverse clinical signs. However, high inclusion (6%) posed risks, including mild hepatic stress and, more critically, signs of immune dysregulation, such as significant eosinophilia, which suggests a potential for allergic sensitization. Therefore, while CPH shows promise, its long-term safety has not yet been established.

Future studies, ideally extending 3–6 months, are essential to evaluate chronic effects on liver function, immune modulation, and potential allergenicity to define optimal and safe inclusion levels for commercial pet food applications.

### Antioxidant efficacy in shelf-life simulation

**Figure 2:**
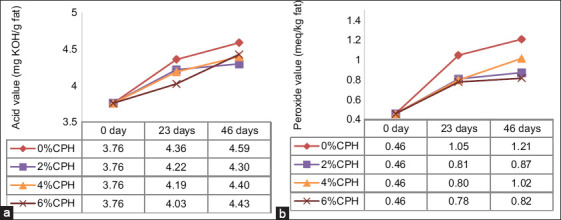
Lipid oxidation stability of diets supplemented with cricket protein hydrolysate at different inclusion levels (0%, 2%, 4%, and 6%) during accelerated storage. Panels show (a) acid value (mg potassium hydroxide/g fat), indicating free fatty acid content, and (b) peroxide value (meq O_2_/kg fat), indicating primary oxidation products, measured after 0, 23, and 46 days of storage at 55°C. Data are presented as mean ± standard error of the mean (n = 3). Within a time point, means with no common letter (a, b, and c) are significantly different (p < 0.05).

The antioxidant efficacy of CPH in mitigating lipid oxidation was evaluated through AV and PV analyses during accelerated shelf-life testing (Figure 2). Over 46 days of storage (equivalent to ~12 months), the control diet (0% CPH) exhibited a progressive increase in AV (3.760–4.590 mg KOH/g oil) and PV (0.460–1.210 meq peroxide/kg), indicating pronounced primary lipid oxidation.

In contrast, the 6% CPH-supplemented diet significantly reduced AV and PV by 3.48% and 33.00%, respectively (p < 0.05), demonstrating dose-dependent antioxidant activity. These results are consistent with those of studies on insect-derived hydrolysates, where enzymatic hydrolysis releases bioactive peptides capable of scavenging free radicals and chelating pro-oxidative metal ions [33, 34].

The antioxidant properties of CPH are attributed to its sulfur-containing amino acids (e.g., methionine and cysteine) and aromatic residues (e.g., tyrosine and tryptophan), which neutralize free radicals and disrupt lipid peroxidation cascades. This hypothesis is supported by recent research on hydrolysates from a different cricket species (*Gryllus bimaculatus*), where low-molecular-weight (<3 kDa) peptide fractions rich in hydrophobic amino acids (e.g., valine and leucine) exhibited strong radical scavenging and metal-chelating capacities [35]. The presence of similar low-molecular-weight peptides in CPH is likely a key contributor to its antioxidant activity.

Despite these positive findings, the study has two main limitations. First, the reliance on AV and PV limits insight into secondary oxidation products (e.g., aldehydes and ketones), which can reduce hydroperoxide levels while masking oxidative degradation [36]. Second, while the 4%–6% CPH inclusion demonstrated significant lipid stability, the absence of a direct comparison to industry-standard preservatives (e.g., butylated hydroxyanisole and tocopherols) means that its relative efficacy is unquantified.

Therefore, future studies should incorporate a more comprehensive assessment of oxidation, such as thiobarbituric acid reactive substances or volatile aldehyde assays, and include direct comparisons against common synthetic and natural antioxidants to better position CPH within the landscape of pet food preservatives.

## CONCLUSION

This study demonstrated that CPH is a promising multifunctional ingredient for canine diets, capable of improving both sensory appeal and functional quality. A 2% inclusion level significantly enhanced palatability, with a 57% increase in feed intake over the control and resulted in the highest BW gain (+1.17 kg) without adverse biochemical or hematological changes. Antioxidant efficacy increased with CPH concentration, with the 6% diet reducing PVs by 33% after 46 days of accelerated storage, suggesting potential use as a natural preservative for lipid-rich formulations.

From a practical standpoint, moderate CPH inclusion (2%–4%) offers a sustainable alternative protein source that supports feed acceptance, provides high protein bioavailability, and can extend shelf life without reliance on synthetic antioxidants. Its inherent antioxidant peptides, hydrophobic amino acids, and sulfur-containing residues add functional value beyond basic nutrition, aligning with current trends toward clean-label and environmentally conscious pet food products.

The primary strength of this study lies in its comprehensive evaluation, integrating amino acid profiling, palatability trials, nutrient digestibility, metabolic and immunological assessments, and shelf-life testing under accelerated storage conditions. The use of standardized palatability protocols and clinically relevant biochemical monitoring enhances the reliability and translatability of the findings for the commercial pet food industry.

However, several limitations must be acknowledged. The 30-day trial duration does not address long-term safety, particularly regarding hepatic adaptation, immune modulation, and allergenic potential at higher inclusion levels. The antioxidant assessment was limited to primary oxidation markers (AV and PV) and did not benchmark CPH efficacy against established natural or synthetic preservatives. In addition, the absence of tryptophan and certain essential amino acids indicates the need for complementary protein blending to achieve complete nutritional adequacy.

Future research should focus on long-term feeding trials (≥3–6 months) to assess chronic effects, immune tolerance, and allergenic risk, particularly in sensitive breeds. Optimization strategies such as encapsulation or flavor masking should be explored to mitigate bitterness at higher inclusion rates, enabling greater antioxidant benefits without compromising palatability. Comparative studies against common preservatives will further define CPH’s competitive position as a functional additive.

CPH represents a sustainable, functional, and nutritionally valuable ingredient for canine diets when used at moderate inclusion levels. By combining palatability enhancement, nutritional benefits, and antioxidant capacity, it offers significant potential for advancing eco-friendly, high-performance pet food formulations. Strategic formulation adjustments and further validation can position CPH as a commercially viable alternative protein and natural preservative in the premium pet food sector.

## AUTHORS’ CONTRIBUTIONS

NS: Collected samples and conducted the experiments. CB: Provided technical assistance during the experimental procedures. KP: Designed the study, collected samples, and revised the manuscript. All authors have read and approved the final version of the manuscript.
